# Knowledge, attitude, practice, and predictors of female genital mutilation in Degadamot district, Amhara regional state, Northwest Ethiopia, 2018

**DOI:** 10.1186/s12905-020-01041-2

**Published:** 2020-08-14

**Authors:** Gedif Melese, Mulugeta Tesfa, Yewbmirt Sharew, Tsegaye Mehare

**Affiliations:** 1 Department of public health, College of Health Sciences, Debre Markos University, Debre Markos, Ethiopia; 2 Departments of Midwifery, College of Health Sciences, Debre Markos University, Debre Markos, Ethiopia; 3 Department of Biomedical science, Collage of medicine and health science, Dilla University, Dilla, Ethiopia

**Keywords:** Degadamot district, Female genital mutilation, Knowledge, Attitude, Practice

## Abstract

**Background:**

Female genital mutilation is defined as all procedures that involve partial or total removal of external female genitalia, or other injuries to the female genital organs for cultural and religious purposes. In Ethiopia, the prevalence of female genital mutilation practice was 70.8% according to Ethiopian demographic and health survey 2016. This practice is against females’ reproductive health rights with many serious consequences in physical, mental, social and psychological makeup. Therefore, this study aimed to assess knowledge, attitude, practice, and predictors of female genital mutilation in Degadamot district.

**Methods:**

A community-based cross-sectional study design was conducted. Three hundred twenty-five mothers who had under 5 years old female children were selected using systematic random sampling from seven kebeles of Degadamot district. Data were collected using an adapted semi-structured face to face interview questionnaire. Data were entered into Epi-data version 3.1 and then exported to SPSS version 20 for analysis. Logistic regression analysis with 95% confidence intervals was carried out to determine the associations between predictor variables and outcome variables.

**Result:**

The finding of this study revealed that 56.6% of mothers had good knowledge about female genital mutilation and 54.2% of participants had a favorable attitude about female genital mutilation. 70.8% of under 5 years old female children’s had female genital mutilation. Marital status AOR = 7.19(95%CI3.22–16.03), monthly income AOR = 1.97(95% CI 0.26–3.81), custom AOR = 2.13(95% CI 1.20–3.78), belief AOR =2.47(95% CI 1.39–4.39), value AOR = 0.37(95% CI 0.22–0.63), and attitude AOR = 24.4(95% CI 20.01–34.76) towards female genital mutilation had significant association with female genital mutilation practice.

**Conclusion:**

Prevalence of FGM practices among female children of under 5 years of age was found to be high as compared to the national level (64%). 56.6% of mothers had good knowledge about FGM. The majority of the women had a favorable attitude to keep FGM practice among their under 5 years old daughters. Marital status, monthly income, custom, belief, value, and attitude had a significant association with FGM practice.

## Background

Female Genital Mutilation (FGM) is all procedures that involve partial or total removal of the external female genitalia for non-medical reasons [1]. The practice of female genital mutilation/circumcision is dated back to ancient times [2]. Female circumcision has existed for over 4,000-5,000 years originating in a period predating God’s covenant with Abraham to circumcise his people. Even if, there is no definitive evidence documenting when or why this ritual begun some theories suggest that FGM began in Egypt and was frequently performed by the ancient cultures of the Phoenicians, Hittites, and ancient Egyptians [3, 4]. Worldwide, in several countries FGM performed for different cultural reasons such as maintain the cleanliness of the vestibule by cutting secretory parts of the genitalia, discouraging promiscuity, aesthetic reasons, safeguarding and proof of virginity, and a prerequisite for honorable marriage [5, 6]. In Ethiopia, girls who are not circumcised are considered as “promiscuity” as a result, have less chance of getting married [7]. The prevalence report estimated that more than 125 million girls and women have been subjected to FGM practice [8]. Two hundred million girls and women in the world are estimated to have undergone FGM, and another 15 million girls are at risk of experiencing it in the high prevalence countries [3, 9]. Despite a high level of knowledge regarding the complications of FGM and awareness of the global campaign against it, the prevalence of FGM in Africa countries such as Somali, Guinea, Mali, Djibouti, Sudan, and Egypt’s is high [10, 11]. In Ethiopia, the prevalence of FGM was 80% according to Ethiopia Demographic Health Survey (EDHS) 2000 [12], 75% according to EDHS 2005 [13], and 70.8% according to EDHS 2016 [14]. Concerning regional states in Ethiopia, the highest prevalence of FGM was found in Afar, Somali, Hadya, and Wolayta but less prevalent in Gambella and Tigray [13, 15]**.** According to EDHS 2005, the prevalence of FGM in the Amhara region was 74% [16] while the cross-sectional study in Lejet kebele, Dembecha woreda, Amhara region in 2014 report that 94% women and 34.2% of under five children were circumcised [17].

FGM practice is against females’ reproductive health rights with many serious consequences in physical, mental, social and psychological makeup. Therefore, this study aimed to assess knowledge, attitude, practice, and predictors of female genital mutilation in Degadamot district.

## Methods

### Study design and setting

A community-based cross-sectional study design was conducted. The study was conducted in Degadamot district, Amhara regional state, Ethiopia. Degadamot is located on 409 Kilometer from Addis Ababa, capital city of Ethiopia. The total population of Degadamot district is 181,222. Reproductive age woman constitutes of 42,732. The total area of Degadamot district is 833.23km^2^, location =11^0^4^,^ 60^″^ North latitude and 37^0^24^′^ 59^″^East longitude [18, 19]. This district has 33 rural Kebeles.

### Operational definition

In this study, “knowledgeable” was defined as a score greater than or equal to a mean value of 5.8 from knowledge measuring ten questions of FGM. “Favorable attitude”: mothers score greater than or equal to mean value of 29.25 from attitude measuring ten questions of FGM classified as favorable attitude. “Practice”: respondents were classified as having FGM practice when there is at least one female daughter exposed to genital mutilation practice among under 5 years old female children in the family. “Belief”: mental acceptance of acclaim as truth regardless of may or may not be supported by religion. “Custom”: frequently repetition of the same standard, value and behavior in ordinary manner. “Value”: accepting of rule, standard and behavior for a given community to have benefit.

### Sample size and sampling procedure

**Fig. 1:**
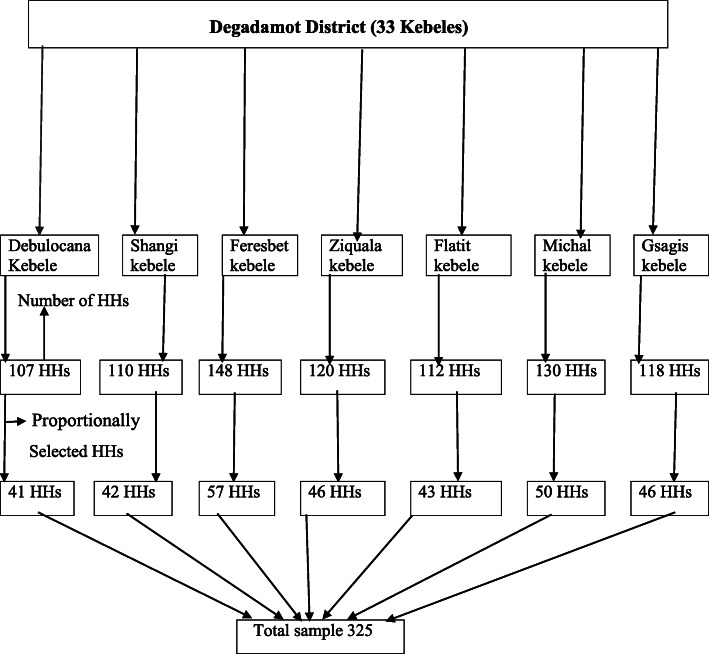
Schematic presentation of the sampling procedure

The sample size was determined using single population proportion formula from Ethiopian demographic and health survey study, 2016(74%) at 95% confidence interval with a marginal error of 5 and 10% non-response rate, the total sample size was 325 mothers. The composition of the 33 kebeles in terms of ethnicity as well as religion is similar. From 33 Kebeles, seven kebeles (Debulocana, Shangi, Feresbet, Ziquala, Flatit, Michal and Gsagis) were selected randomly through the lottery method. In each of the selected kebeles, two points were identified to start selecting respondents by way of Households (HHs) and a house to house search for the eligible candidate until the required sample size achieved. In each household, the mother was selected as the study subject. Allocation of the desired number of households in each selected kebeles was done based on the number of households reported by respective kebeles (Fig. 1). The first household selected with lottery method, then, every 2nd household was included by a systematic random sampling method.

### Data collection

A face to face-administered structured questionnaire was used to collect the data (Additional File 1). The questionnaire was prepared in English and then translated into Amharic, a local language. The questionnaire consisted of items assessing socio-demographic characteristics, knowledge, attitude, and practice of FGM. Seven experienced high school teachers for data collection and two experienced Master of Science (MSc) graduate health professionals for supervision were recruited. One day training was given for data collectors and a pre-test was done on 32 samples out of the main study area. During the pre-test, the questionnaire was assessed for its clarity, understandability, completeness, and time consumption. On each day until the end of the data collection period, trained data collectors were collected the data and submitted filled questionnaire to their respective supervisors daily. Subsequently, data were checked for completeness, accuracy, and consistency accordingly.

### Inclusion and exclusion criteria

Inclusion criteria: All volunteer mothers who respond and had female children of under 5 years of age.

Exclusion criteria: Mothers who were seriously sick at the time of the interview.

### Data analysis

After coding data entered and cleaned using EPI-DATA version 3.1 then exported to Statistical Package for Social Science (SPSS) version 20 for further analysis. Descriptive statistics were calculated for each variable. Bivariate logistic regression analysis was done to make a decision whether there is an association between dependent variable and independent variable and then, to select nominee variables for multivariate logistic regression. Variables with *p*-values of up to 0.05 in the bivariate logistic regression analysis were identified and fitted to the multiple logistic regression analysis to identify the independent effects of each variable to the outcome variable. The odds ratio with a 95% confidence intervals (CI) was calculated to distinguish the occurrence and strength of associations, and statistical significance was affirmed if *p* < 0.05.

## Results

### General characteristics of study participants

Hundred percent of the study participants were Orthodox Christians in religion and Amhara in ethnicity. Moreover, 245(75.4%) study participants were married whereas in a profession almost all (98.5%) of study participants were farmers. Regarding monthly income, slightly more than one-half (54.5%) of study participants had less than 300 Ethiopia birrs. Almost two-thirds (70.8%) of participants were unable to read and write (Table 1).

**Table 1:** General characteristics of study participants in Degadamot district

Variable	Frequency	Percentage (%)
Age in years		
15–24	17	5.2
25–34	176	54.2
≥ 35	132	40.6
Marital status		
Married	245	75.4
Divorce	43	13.2
Widowed	37	11.4
Income level		
< 300 birr	177	54.5
≥ 300 birr	148	45.5
Educational status		
Cannot read and write	230	70.8
Can read and write (without formal education)	60	18.5
Completed primarily education	31	9.5
Completed from 9th_−12_th	4	1.2
Occupational status		
Farmer	320	98.5
Merchant	5	1.5
Religion		
Orthodox Christian	325	100
Ethnicity		
Amhara	325	100

### Knowledge of female genital mutilation

**Fig. 2:**
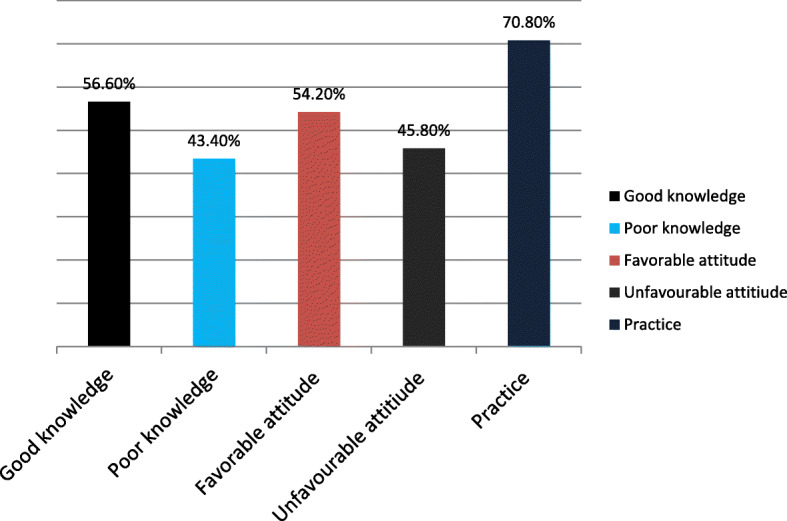
Knowledge, attitude, and practice of the study participants for female genital mutilation in Degadamot District

One hundred eighty four (56.6%) mothers had good knowledge of female genital mutilation. 184 (56.6%) mothers knew FGM can increase transmission of HIV/AIDS and decrease sexual pleasure 184 (56.6%) (Fig. 2) and (Table 2).

**Table 2:** Knowledge of study participants about genital mutilation in Degadamot district

knowledge question	Yes (percentage)	No (percentage)
Do you know FGM has psychologically harmful?	211 (64.9%)	114 (35.1%)
Do you know FGM can decrease sexual pleasure?	184 (56.6%)	141 (43.4%)
Do you know FGM has a problem with health?	183 (56.3%)	142 (43.7%)
Do you know FGM has a contribution to HIV / AIDS transmission?	184 (56.6%)	141 (43.4%)
Do you know FGM brings complication during delivery?	173 (53.2%)	152 (46.8%)
Do you know FGM is harmful traditional practice?	209 (64.3%)	116 (35.7%)
Do you know the FGM has scar formation effect?	208 (64%)	117 (36%)
Do you know FGM has a negative side effect on health?	168 (51.7%)	157 (48.3%)
Do you know FGM is forbidden in the law?	188 (57.8%)	137 (42.2%)
Is there any other health problem concomitant with FGM?	130 (40%)	195 (60%)

### Attitude of female genital mutilation

The attitude of mothers towards FGM practice was assessed nevertheless, more than half of the (54.2%) participants had favorable attitudes and 149(45.8%) participants had an unfavorable attitude (Fig. 2) and (Table 3).

**Table 3:** Attitude of study participants about FGM practice in Degadamot districts

Attitude question	Strongly agree	Agree	Neutral	Disagree	Strongly disagree
Do you support FGM?	35 (10.8%)	59 (18.2%)	24 (7.4%)	155 (47.7%)	51 (15.7%)
Does FGM can protect virginity of female?	43 (13.2%)	97 (29.8%)	18 (5.5%)	142 (43.7%)	25 (7.7%)
Do you think uncircumcised females are not faithful	49 (15.1%)	95 (29.2%)	21 (6.5%)	111 (34.1%)	49 (15.7%)
Does uncircumcised female virginity can’t easily rupture during first time sex?	11 (3.4%)	87 (26.8%)	13 (4%)	142 (43.7%)	25 (7.7%)
Do you think uncircumcised females have decrease sexual feeling?	12 (3.7%)	95 (29.2%)	10 (3.1%)	47 (14.5%)	162 (49.5%)
Do you think FGM is good practice?	22 (6.8%)	95 (29.2%)	14 (4.3%)	167 (51.4%)	27 (8.3%)
Do you think uncircumcised female has problem During child birth?	58 (17.8%)	80 (24.6%)	11 (3.4%)	167 (51.4%)	27 (8.3%)
Will you voluntarily circumcise if you have daughter?	28 (8.6%)	155 (47.7%)	19 (5.8%)	103 (31.7%)	20 (6.2%)
Do you think uncircumcised female calls as a maid in societies?	23 (7.1%)	!72 (52.9%)	5 (1.5)	90 (27.7%)	35 (10.8%)
Do you agree with FGM continuity for the future?	22 (6.8%)	147 (45.21)	11 (3.4%)	136 (41.8%)	9 (2.8%)

### Female genital mutilation practice

Among under 5 years old female children’s in Degadamot districts, 230(70.8%) had a practice of FGM. In this study circumcision was commonly practiced at 7th day of postnatal age (11.5%), 8th day of postnatal age (86.5%), and 9th day of postnatal age (2%) (Table 4) and (Fig. 2). Commonly Orthodox Christian religion follower’s in Ethiopia baptized delivered mother with holy water around day 7th, 8th, 9th and FGM takes place accompanied with it.

**Table 4:** Genital mutilation practice for under 5 years old daughters in Degadamot district

Practice based question	Yes (percentage)		No (percentage)
Is FGM performed for your under 5 years old daughters	230 (70.8%)		95 (29.2%)
Practice based question	7th day	8th day	9th day
When circumcision takes place after birth?	37 (11.5%)	281 (86.5%)	7 (2%)

### Predictors of knowledge of female genital mutilation

Income of respondents, marital status, belief, custom, and value had a significant association with knowledge and attitude about female genital mutilation. Married mothers were 5.64 times more likely to had good knowledge about female genital mutilation than widowed mothers (AOR = 5.64 Cl: 1.78, 10.95). Those mothers who had a monthly income of ≥300 birrs were 2.15 times more likely to had good knowledge than those mothers who had < 300 birrs monthly income (AOR = 2.15Cl: 1.22,3.73). Belief and custom had association with knowledge about female genital mutilation (AOR = 2.74Cl: 1.71, 4.27) and (AOR = 3.14 Cl: 1.82, 5.43) respectively. Values had association with knowledge about female genital mutilation (AOR = 2.89 Cl: 1.68, 4.98) (Table 5).

**Table 5:** Bivariate and multivariate logistic regression analyses of factors associated with FGM knowledge and attitude in Degadamot district

Characteristic	Knowledge		AOR (95% CI)	*p*-value	Attitude		AOR (95%CI)	*p*-value
	Good	Poor			favorable	Unfavorable		
Income								
≥ 300birr	113	64	2.15 (1.24,3.73)	0.006	78	99	5.52 (3.18,9.56)	0.031
< 300birr	71	77	1		83	65	1	
Marital status								
Married	161	84	5.64 (1.78,10.94)	0.001	102	143	2.33 (1.85,4.32)	0.002
Divorced	14	29	1.42 (0.52,3.87)	0.49	29	14	0.45 (0.17,1.36)	0.692
Widowed	9	28	1		30	7	1	
Belief								
No	124	94	2.74 (1.72,4.27)	0.001*	115	49	4.77 (2.75,8.26)	0.001
yes	60	47	1		105	56	1	
Custom								
No	123	55	3.14 (1.82,5.43)	0 .001*	63	115	3.99 (2.31,6.89)	0.001
yes	61	86	1		98	49	1	
Value								
No	130	54	0.28 (0.16,0.49)	0.001*	64	123	2.12 (1.22,3.66)	0.007
yes	54	87	1		97	41	1	

### Predictors of attitude of female genital mutilation

Married mothers were 2.33 times more likely to had unfavorable attitude towards female genital mutilation than widowed (AOR = 2.33Cl: 1.85, 4.32). Those mothers whose monthly income ≥300 birrs were 5.52 times more likely to had a unfavorable attitude than those mothers whose monthly income < 300 birrs (AOR = 5.25 Cl: 3.18, 9.65). The other significant associated factor with the attitude of mothers’ is belief (AOR = 4.77Cl: 2.75, 8.26). In this study custom and value also had significantly associated factors with attitude of mothers towards female genital mutilation (AOR = 3.99 Cl: 2.31, 6.89) and (AOR = 2.12 Cl: 1.23, 3.66) respectively (Table 5).

### Predictors of female genital mutilation practice

Marital status, monthly income, custom, belief, value, and attitude had a significant association with FGM practice. Married mothers were 7.19 time more likely to had practiced female genital mutilation for their under 5 years old female children’s than widowed practiced of female genital mutilation for their under 5 years old female children’s (AOR = 7.19 Cl: 3.22, 16.03). Those mothers whose monthly income of ≥300 birrs were 1.97 times more likely to had practiced female genital mutilation than those who had < 300 birrs monthly income (AOR = 1.97Cl:0.25, 0.81). Custom and belief also significantly associated factors to the attitude of mothers towards FGM (AOR = 2.13Cl: 1.20, 3.78) and (AOR = 1.47Cl: 1.39, 4.39) respectively. This study also showed value had significant associated factors for FGM practice (AOR = 0.37Cl: 0.22, 0.63). Those mothers who had unfavorable attitudes toward FGM were 24.4 times more likely to had a practice of female genital mutilation for their under 5 years old female children than mothers with favorable attitude (AOR = 24.4 Cl: 20.01, 347.59) (Table 6).

**Table 6:** Bivariate and multivariate logistic regression analyses of factors associated with FGM practice in Degadamot district

Characteristics	FGM		COR (95%CI)	AOR (95%CI)	*p*-value
	Yes	No			
Marital status					
married	200	45	8.205 (3.882–17.343)	7.19 (3.223–16.03)	0.001*
divorced	17	26	1.207 (0.486,3.001)	0.483 (0.171,1.369)	0.171
widowed	13	24	1	1	
Believe					
no	139	32	3.007 (1.822,4.962)	2.472 (1.391,4.394)	0.003
yes	91	63	1	1	
Income					
> 300birr	92	56	2.154 (1.324,3.504)	1.97(0.258,3.810)	0.027
< 300 birr	138	39	1	1	
Value					
no	150	37	2.939(1.794,4.815)	0.374(0.222,0.632)	0.01*
yes	80	58	1	1	
Custom					
no	141	37	2.483(1.521,4.055)	2.133 (1.203,3.7830)	0.001*
yes	89	58	1	1	
Knowledge					
good	92	138	1.38 (0.832,2.27)	1.21 (0.134,2.212)	0.140
poor	31	64			
Attitude					
unfavorable	93	148	25.5 (20.155,349.47)	24.4 (20.008,347.59)	0.001*
favorable	2	82	1	1	

*p<0.05

## Discussion

### Knowledge of female genital mutilation

56.6% of study participants had knowledge about the harmful effect of FGM which is slightly good as compared to the studies conducted on mothers’ knowledge about female genital mutilation in the Amhara region, Dembecha woreda and Oromia region [20, 21]. The possible rationalization for this variation could be due to time interval and better health education provided by health extension workers to the study participants in the current study area. The other justification for differences might be due to differences in place of the studies that might be explained by different strategies in promoting and creating awareness about the terrible health consequences of FGM and cultural differences in study participants especially in Oromia region where most of the people are Muslims. However, the study done in Jijiga showed that mother’s knowledge about the terrible effect of FGM was higher. This might be due to the fact that massive governmental and nongovernmental intervention in Somalia region to create awareness and stop the practice [22]. Hence, the mother’s knowledge on the terrible effect of FGM is very poor in this study area that needs intervention from health professionals, government and other concerned bodies.

### Attitude of female genital mutilation

54.2% of study participants had a favorable attitude against female genital mutilation. It was inconsistent with the study done in Amhara region, Oromia region, and Gambia [20, 21, 23]. The possible explanation for this difference might be due to the time gap and socio-demographic background different particularly with Gambia. Another possible rationalization for this difference might be religious by which all of the study participants in the current study were orthodox Christians.

### Predictors of knowledge and attitude for female genital mutilation

Income of respondents, marital status, belief, custom, and value had a significant association with knowledge and attitude of mothers about the terrible effect of female genital mutilation. Those mothers who were married had good knowledge and unfavorable attitude about the terrible effect of female genital mutilation than widowed. The possible justification for this association was those mothers who were married had experience terrible effects especially, during sexual practice how it is painful and lengthen labor with its horrible consequences, even if, no other study which supports it.

Mothers whose monthly income ≥300 Ethiopian birrs were more likely to had good knowledge and unfavorable attitude towards the effect of FGM than whose monthly income < 300 Ethiopian birrs. This finding is consistent with studies conducted in Jimma zone, Southwest Ethiopia [24], Maryland USA [25], WHO [26]. This might be due to the fact that economically well-off families were more likely to expose to digital and non-digital educational programs and acquired knowledge to develop unfavorable attitude FGM. However, this observation was contrary to most studies’ findings which showed that the knowledge, attitude, and practice of FGM is independent of economic status. Moreover, the knowledge and attitude of mothers about FGM was significantly predicted by belief, custom, and value. Comparable results were seen by a study in Sudan [3, 27]. The possible justification for this might be an individual’s belief, custom and values are preloaded social and cultural identities as well as important factors for having a negative attitude towards FGM and acquire knowledge.

### Predictors of female genital mutilation practice

70.8% of female children age fewer than 5 years old had genital mutilation in Degadamot woreda which was lower than the study conducted in Eastern Sudan [28] and Kenya [26]. The possible explanation for this difference could be due to the difference in study participants sample size and socio-demographic difference. Another possible rationalization for this difference might be the difference in culture and religion because in this study all the study participants were orthodox Christians as compared to Sudanese who were mostly Muslims. However, this study finding was lower than the study conducted in Dembecha woreda, Amhara region [21]. The possible reason for this difference could be the time gap otherwise socio-cultural and religious aspects were almost similar.

Marital status, monthly income, custom, belief, value, and attitude had a significant association with female genital mutilation practice. Those married women were more likely to had practiced female genital mutilation for their under 5 years old female children’s than widowed women practiced female genital mutilation for their under 5 years old female children’s. However, married women had experienced pain particularly during sexual practice and prolong labor with its horrible consequences, their daughters might be circumcised to avoid promiscuity and be a candidate for honorable marriage. Those women who had a monthly income of ≥300 birrs were more likely to had practiced female genital mutilation than those who had less than 300 birrs monthly income. This finding was contrary to the studies conducted in Maryland USA [25], WHO [26]. This difference might be due to study sitting and culture. However, most studies’ findings showed that the knowledge, attitude, and practice of FGM are independent of economic status.

Custom, belief, and value of women also other important significant factors associated with female genital mutilation practices. It was in line with a study done in Jimma zone [24] and Eastern Ethiopia [29]. FGM is performed in line with tradition and social norms and to uphold their status and honor of the entire family. Belief, custom, and values are environmentally acquired indigenous identities that regulate individual’s perception towards terrible effect FGM practices [29, 30]. Attitude was also another significantly associated factor with FGM practice. Women who had unfavorable attitude against FGM were more likely to practice FGM on their daughters than women who had a favorable attitude against FGM. It was consistent with study done on Somali region, particularly female participants [29]. The possible justification for this might be the practice of FGM is the result of poor knowledge and unfavorable attitude against the harmful effects of FGM. Women with good knowledge of the harmful effects of FGM more likely not to support the practice and in turn not engage the practice. This means educate women about the harmful effects of FGM will aid them to build up constructive attitude to impede the practice.

## Conclusion

The prevalence of FGM practices among female children of under 5 years of age was found to be high as compared to the national level (64%). Most women had good knowledge about FGM but majority of them had a favorable attitude to keep FGM among their daughters. Marital status, monthly income, custom, belief, value, and attitude had a significant association with FGM practice.

### Recommendations

Government has to take a strong legal measurement of female genital mutilation. Regional Health Bureau and Zonal Health Offices must plan health education program about the ill health effects of FGM and provide community education program. Health profession has to give attention during antenatal care, postnatal care, and other maternal health services in creating awareness about ill health effects of FGM. Lastly, further qualitative research is recommended.

## Data Availability

The datasets generated and/or analyzed during the current study are available from the corresponding author on reasonable request.

## Abbreviations

AOR: Adjust odds ratio
COR: Crude odds ratio
EDHS: Ethiopia Demographic Health Survey
FGM: Female genital mutilation
KAP: Knowledge attitude and practice
HHs: House holds
SPSS: Statistical package for social science

## References

[1] EN: Female genital mutilation. Report of a research methodological workshop on estimating the prevalence of FGM in England and Wales. London. 2012.

[2] Teixeira ALLM (2016). Estimating the prevalence of female genital mutilation in Portugal. Public Health.

[3] Werunga DS (2017). Transformation of female circumcision among the Kipsigis of Bomet County: Kenya; 1945–2014.

[4] Van Baelen L, Ortensi L, Leye E. Estimates of first-generation women and girls with female genital mutilation in the European Union, Norway and Switzerland. The European Journal of Contraception & Reproductive Health Care. 2016;21(6):474-82. 10.1080/13625187.2016.1234597 27652839

[5] Gale T: Encyclopedia of bioethics; , volume 1. In*.* Edited by Stephen G. Post eic, vol. volume 1, 3rd ed edn.

[6] Saleem RA, Othman N, Fattah FH, Hazim L, Adnan B (2013). Female genital mutilation in Iraqi Kurdistan: description and associated factors. Women Health.

[7] SA: Activity Report of Ethiopian Women Layers association. In*.*, vol. 10; 2000: 21.

[8] Leye E, Powell RA, Nienhuis G, Claeys P, Temmerman M (2006). Health care in Europe for women with genital mutilation. Health Care Women Int.

[9] Abdulcadir J, Margairaz C, Boulvain M, Irion O: Care of women with female genital mutilation/cutting. Swiss Med Wkly 2011, 141(1-2). 10.4414/smw.2011.13137 21213149

[10] Khosla R, Banerjee J, Chou D, Say L, Fried ST (2017). Gender equality and human rights approaches to female genital mutilation: a review of international human rights norms and standards. Reprod Health.

[11] Waigwa S, Doos L, Bradbury-Jones C, Taylor J (2018). Effectiveness of health education as an intervention designed to prevent female genital mutilation/cutting (FGM/C): a systematic review. Reprod Health.

[12] Ethiopian Demographic and Health Survey. In*.*; 2000.

[13] Assefa R: Factors affecting the practice of female genital mutilation of Ethiopian women. Addis Abeba university; 2011.

[14] Mehari LE (2018). The Association of Female Genital Mutilation in sexual behaviors and marriageability, Ethiopia DHS 2016.

[15] Gebremichael T (2002). Female genital mutilation and birth complications, Jijiga town.

[16] Central statistical authority (CSA) and Ethiopian demographic and health survey study. In*.*; 2005.

[17] Moges NA, Mullu G, Gedfew M, Redi M, Molla M, Ayenew S, Fentahun S, Adisie S, Dagnew Z (2015). Knowledge, attitude and practice of women towards female genital mutilation in Lejet Kebele, Dembecha Woreda, Amhara regional state, northwest, Ethiopia, 2014. J Gynecol Obstetr.

[18] Fikre AA, Demissie M (2012). Prevalence of institutional delivery and associated factors in Dodota Woreda (district), Oromia regional state, Ethiopia. Reprod Health.

[19] Wako WG, Kassa DH (2017). Institutional delivery service utilization and associated factors among women of reproductive age in the mobile pastoral community of the Liban District in Guji zone, Oromia, southern Ethiopia: a cross sectional study. BMC Pregnancy Childbirth.

[20] Belda S, Tololu A (2017). Knowledge, attitude and practice of mothers towards female genital mutilation in south west Shoa zone, Oromia region, Ethiopia. MOJ Public Health.

[21] Moges NA, Mullu G, Gedfew M (2015). Attitude and practice of women towards female genital mutilation in Lejet Kebele, Dembecha Woreda, Amhara regional state, northwest, Ethiopia, 2014. J Gynecol Obstet.

[22] Hussein MA, Abdi AA, Mohammed M (2013). Knowledge, attitude and practice of female genital mutilation among women in Jigjiga town, Eastern Ethiopia. Gaziantep Med J.

[23] Kaplan A, Hechavarría S, Martín M, Bonhoure I (2011). Health consequences of female genital mutilation/cutting in the Gambia, evidence into action. Reprod Health.

[24] Argaw A, Fisseha N: Prevalence of female genital mutilation and attitude of mothers towards it in serbo town. Ethiop J Health Sci 2002, 12(2): 69-68.

[25] Carr D (1997). Female genital cutting. Findings from the Demographic and Health Surveys program.

[26] Organization WH (2000). A systematic review of the health complications of female genital mutilation including sequelae in childbirth.

[27] Islam MM, Uddin MM. Female circumcision in Sudan: future prospects and strategies for eradication. Int Fam Plan Perspect. 2001;27:71–6.

[28] El Dareer A (1983). Complications of female circumcision in the Sudan. Trop Dr.

[29] Abathun AD, Sundby J, Gele AA (2016). Attitude toward female genital mutilation among Somali and Harari people, Eastern Ethiopia. Int J Women’s Health.

[30] Fund UNCs, Gupta GR. Female genital mutilation/cutting: a statistical overview and exploration of the dynamics of change. Reprod Health Matters. 2013;21:184–90.

